# RESIDENTS’ PERSPECTIVES ON PREFERENCES: EXPLORING HOW NURSING HOME RESIDENTS EXPRESS AND ACHIEVE PREFERENCES

**DOI:** 10.1093/geroni/igae098.1099

**Published:** 2024-12-31

**Authors:** Laura Block, Caroline Madrigal, Tonya Roberts

**Affiliations:** University of Wisconsin-Madison, Madison, Wisconsin, United States; VA Boston Healthcare System, Brockton, Massachusetts, United States; University of Wisconsin - Madison, Madison, Wisconsin, United States

## Abstract

Providing choice and accommodation of preferences is fundamental to delivery of person-centered care in nursing homes (NH). Studies indicate variability in NH residents’ preferences around a range of daily care routines and activities (i.e., involvement of family, meal choices, morning or nighttime routines) and the widespread challenges NHs face in meeting preferences. Yet, few studies have examined NH resident perspectives on their role as a key player in communicating their preferences. This qualitative study is part of a broader mixed methods exploration of the relationship between preference-aligned care and quality of life. A subset of residents (N=11) was selected for semi-structured interviews to examine resident perspectives on navigating preferences in daily life. Interviews were coded inductively, with thematic analysis guiding abstraction and interpretation. Residents described a range of degree of preference expression, from voicing strong preferences for or against care/activities; deferring to staff; or being uncertain of a preference. Residents shared multiple strategies for meeting their preferences, including communicating with new staff, relying on family, escalating requests, and adjusting to the NH environment. Residents also described staff-led strategies of documenting and sharing their preferences and splitting the work into parts, which often required resident collaboration and input. The importance of consistency in staffing and developing relationships with staff was underscored. Future work should examine the potential role health, cognitive, and functional capacity play in shaping residents’ preference expression.

